# The neurological complications of chikungunya virus: A systematic review

**DOI:** 10.1002/rmv.1978

**Published:** 2018-04-19

**Authors:** Ravi Mehta, Patrick Gerardin, Carlos Alexandre Antunes de Brito, Cristiane Nascimento Soares, Maria Lucia Brito Ferreira, Tom Solomon

**Affiliations:** ^1^ National Institute for Health Research Health Protection Research Unit in Emerging and Zoonotic Infections University of Liverpool Liverpool UK; ^2^ INSERM CIC1410 Centre Hospitalier Universitaire de la Réunion Saint Pierre Réunion France; ^3^ UM 134 PIMIT CNRS 9192, INSERM U1187, IRD 249 Université de la Réunion, CHU, CYROI Saint Pierre Réunion France; ^4^ Department of Infectious Diseases Federal University of Pernambuco Recife Brazil; ^5^ Department of Neurology Hospital Federal dos Servidores do Estado Rio de Janeiro Brazil; ^6^ Department of Neurology Hospital da Restauração, Avenida Agamenon Magalhães Recife Brazil; ^7^ Department of Neurology Walton Centre NHS Foundation Trust Liverpool UK; ^8^ Institute of Infection and Global Health University of Liverpool Liverpool UK

**Keywords:** acute disseminated encephalomyelitis, chikungunya, complications, congenital infections, encephalitis, Guillain‐Barré syndrome, myelitis, neonatal infection, neurological, optic neuritis, retinitis, uveitis

## Abstract

We performed a systematic review on the neurological complications of chikungunya virus. Such complications are being reported increasingly, owing primarily to the scale of recent epidemics but also to a growing understanding of the virus' neurovirulence. We performed a thorough literature search using PubMed and Scopus databases, summating the data on all published reports of neurological disease associated with chikungunya virus. We appraised the data for each major condition in adults, children, and neonates, as well as evaluating the latest evidence on disease pathogenesis and management strategies. The review provides a comprehensive summary for clinicians, public health officials, and researchers tackling the challenges associated with this important emerging pathogen.

**Abbreviations: Used in main text**
AIDP: acute inflammatory demyelinating polyneuropathy
AMAN: acute motor axonal neuropathy
AMSAN: acute motor and sensory axonal neuropathy
CNS: central nervous system
CSF: cerebrospinal fluid
ECSA: East/Central/South African lineage
GBS: Guillain‐Barré syndrome
MRI: magnetic resonance imaging
PCR: polymerase chain reaction
RNA: ribonucleic acid

**Used in tables only**
ADEM: acute disseminated encephalomyelitis
BL: bullous lesions
CIDP: chronic inflammatory demyelinating polyneuropathy
CN: cranial nerve
CRAO: central retinal artery occlusion
DIC: disseminated intravascular coagulation
EEG: electroencephalogram
HI: haemagglutination inhibition
Isol: viral isolation
MFS: Miller Fisher syndrome
MP: methylprednisolone
PL: prodrome length
PP: days postpartum
RAPD: relative afferent papillary defect
UMN/LMN: upper/lower motor neuron
UR/I: urinary retention/incontinence
VEP: visual evoked potential
WCC: white cell count

## 1 INTRODUCTION

Chikungunya virus is an alphavirus (genus *Alphavirus*, family Togaviridae) that is primarily transmitted to humans by *Aedes* mosquitoes, and occasionally from mother to child. The word “chikungunya” originates from the Makonde language, spoken in Tanzania and Mozambique, meaning “that which bends up”^1^; this refers to the debilitating arthralgia often occurring in the acute phase of infection, along with fever, myalgia, headache, and rash. Although the first outbreaks were described in the 1960s, the virus was not considered a major public health problem until 2004, when it caused explosive outbreaks in the tropics. Severe complications of chikungunya infection, including neurological disease, are being recognised increasingly.

Classically, alphaviruses are described in 2 groups—the “old world” viruses, including Sindbis, O'nyong'nyong, and Ross River viruses, which cause a predominantly arthritic syndrome, and the “new world” viruses, including Eastern, Western, and Venezuelan equine encephalitis viruses, which are responsible for outbreaks of encephalitis.^2^ Chikungunya virus is now recognised as a cause of both arthritic and neurological diseases throughout the tropics.

Dengue and Zika are also arthropod‐borne viruses (arboviruses) that, like chikungunya, are transmitted by *Aedes* mosquitoes but are flaviviruses (genus *Flavivirus*, family Flaviviridae). All 3 arboviruses cause an initial fever‐arthralgia‐rash syndrome and are associated with neurological complications.^3^, ^4^, ^5^ Given increasing reports of cocirculation and coinfection of the 3 arboviruses in the Americas,^6^ the underlying viral aetiology in patients presenting with arbovirus‐associated neurological disease is not always clear. Therefore, understanding the similarities and differences between chikungunya‐, Zika‐, and dengue‐associated neurological diseases is of importance and will be addressed in this review.

We performed a systematic review for evidence of neurological disease associated with chikungunya virus. A well‐designed review was recently published on this topical subject^7^; our review approaches the disease spectrum in a different manner, accounting for the differences. Here, we use a broader search strategy, leading us to consider general features of the virus and disease before individually discussing the different neurological manifestations, the challenges in their diagnosis, sequelae of perinatal infection, and knowledge of the underlying disease mechanisms; we include comparison with Zika and dengue viruses throughout. Where provided, we present all data detailing the clinical information, investigations, management, and outcome for all cases described in the literature.

## 2 METHODS

**Figure 1:**
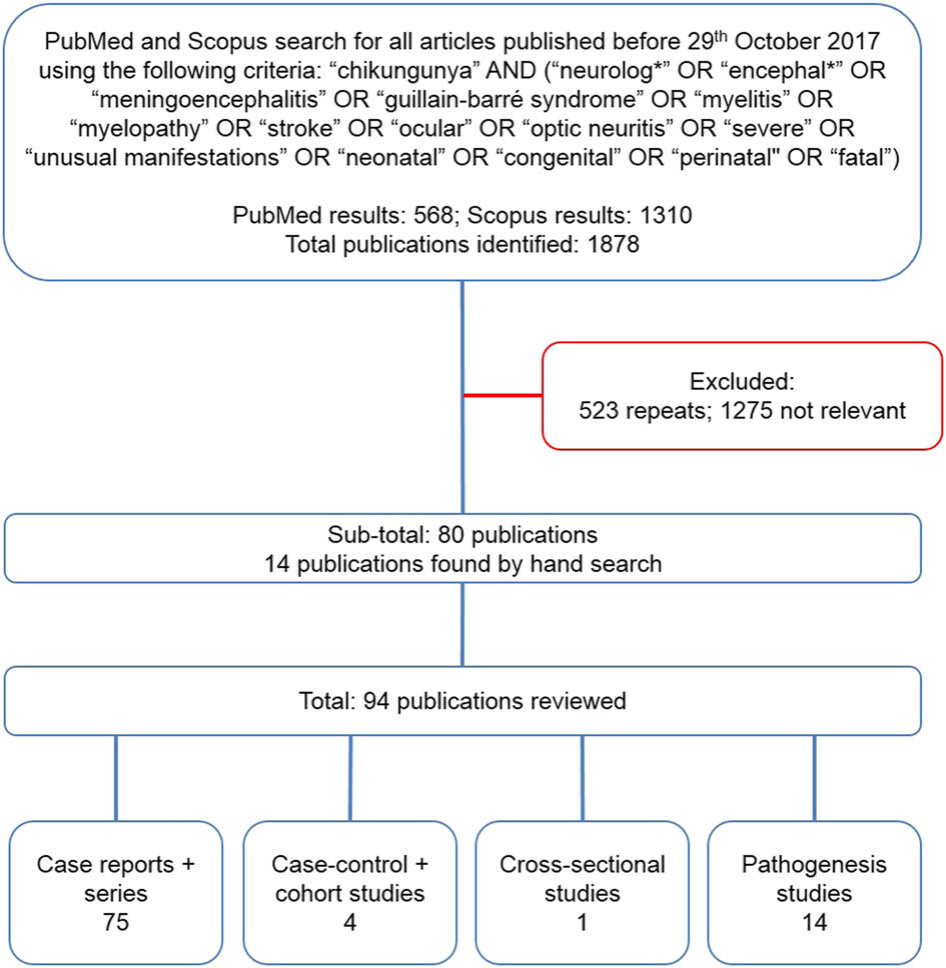
Search strategy to identify publications on neurological complications of chikungunya

We searched PubMed and Scopus for articles published up to 29 October 2017 using the following criteria: “chikungunya” AND (“neurolog*” OR “encephal*” OR “meningoencephalitis” OR “guillain‐barré syndrome” OR “myelitis” OR “myelopathy” OR “stroke” OR “ocular” OR “optic neuritis” OR “severe” OR “unusual manifestations” OR “neonatal” OR “congenital” OR “perinatal” OR “fatal”) (Figure 1). There were no language restrictions. All published studies describing patients with neurological complications of chikungunya were considered eligible for inclusion, comprising case reports and series, and case‐control, cohort, cross‐sectional, and pathogenesis studies. Articles that did not mention neurological complications of chikungunya were excluded. Data extracted included the numbers of patients with neurological complications, and where available, the time between systemic symptoms of infection and neurological disease, cerebrospinal fluid (CSF) findings, a summary of the clinical presentation, the author's diagnosis, treatment given, and outcome. The number of patients described per distinct neurological syndrome and aggregate data on diagnosis, treatment, and outcome were summarised.

## 3 ROLE OF THE FUNDING SOURCE

The funders of the study had no role in study design, data collection, data analysis, data interpretation, or writing of the report. The corresponding author had full access to all the data in the study and had final responsibility for the decision to submit for publication.

## 4 EPIDEMIOLOGY AND LINEAGE

Historical accounts suggest that chikungunya may have caused outbreaks as early as the 18th century,^8^ although the virus and its disease were first isolated and documented respectively in 1952 to 1953, in Tanzania.^9^, ^10^ Since then, 2 lineages, namely West African and East/Central/South African (ECSA), have been shown to circulate in sub‐Saharan Africa in a sylvatic cycle between mosquitoes and non‐human primates.^11^ The first documented human outbreaks were in southern Asia during the 1960s to 1970s^12^, ^13^ and were caused by the Asian strain, a descendent of the ECSA strain.^14^ After decades of low transmission, an ECSA divergent strain re‐emerged in 2004, having undergone 2 successive mutations of its envelope E1 glycoprotein.^15^ This new lineage, renamed the Indian Ocean Lineage spread from Kenya to cause explosive outbreaks throughout the islands of the Indian Ocean, India, and Southeast Asia, affecting millions.^16^ In late 2013, the emergence of the Asian strain was reported in the Caribbean,^17^ marking its first documented appearance in the Americas. It has since rapidly spread throughout 48 American countries and territories, causing over 2 million suspected cases to date.^18^ Of note, local circulation of the ECSA strain has also recently been reported in Bahia state, Brazil.^19^

## 5 DIAGNOSIS

As for other arboviruses, proving that chikungunya has caused neurological disease can be challenging.^2^, ^5^ Traditionally, a causal relationship between microbe and disease was based on Koch's postulates, as follows^20^:

1. The agent must be demonstrable in every case of the disease.
2. The agent is not present in other diseases.
3. After isolation in culture, the agent must be able to produce the disease in experimental animals.
4. The agent can be recovered from the experimental animal.

There are clear limitations to these in modern microbiology. For example, we know now that certain pathogens can cause multiple diseases, and indeed certain diseases can be caused by more than 1 pathogen (postulates 1 and 2). Furthermore, modern technologies such as polymerase chain reaction (PCR) assays have increased our detection rate of certain pathogens over isolation in culture (postulate 3). We therefore adapt these postulates today in studies regarding causality. Neurological disorders associated with arbovirus infection have an added layer of complexity, owing to the different samples used for testing for the presence of the viruses. The strongest evidence of causality comes from demonstrating that the virus is in the central nervous system (CNS), which is most often shown by detecting viral RNA in the CSF by PCR; alternatively, the virus may be cultured. In fatal cases, autopsy material may be positive by PCR. For many patients, the virus has cleared from the CSF by the time they present; in which case, the detection of CSF IgM antibody by enzyme‐linked immunosorbent assay is considered diagnostic. Interpretation is complicated for flavivirus infections because a positive Zika‐IgM test can result from cross‐reactivity of serum containing antibodies against dengue virus.^21^ Because chikungunya is an alphavirus, there is no serological cross‐reactivity with the flaviviruses, making diagnosis more straightforward (in areas where other alphaviruses are not circulating). It is not known for how long virus, RNA, or IgM remains detectable in chikungunya‐associated neurological disease, and whether this differs for the different neurological disorders. By analogy with similar arboviruses, we might expect the virus to be detectable for the first few days of illness, at which point it is replaced by antibody, which remains for weeks to months. Chikungunya virus is more readily detected by PCR or culture in the blood, because of its long and high viraemia; IgM antibody can also be detected in the blood. However, a positive blood test in a patient with neurological disease does not necessarily mean the virus caused the disease; infection may be coincidental, and care must be taken to exclude other possible causes. The virus can also be detected in urine, saliva, semen, and milk,^22^, ^23^, ^24^ but the same caveats apply.

## 6 CLINICAL FINDINGS

Seroprevalence studies have reported a range of asymptomatic rates of chikungunya infection, from 3% to 47%.^25^ In acute symptomatic infection, following an incubation period of approximately 3 days,^26^ there is an abrupt onset of fever, headache, rash, arthralgia, and myalgia, which typically last for 1 to 2 weeks.^27^ After this, seroconversion likely confers lifelong immunity.^28^ As well as neurological manifestations, chikungunya virus is associated with complications of the cardiovascular, renal, respiratory, hepatic, gastrointestinal, and adrenal systems, sometimes collectively referred to as “atypical features.”^29^, ^30^, ^31^ However, a disorder of the nervous system appears to be the most common severe complication of chikungunya infection (Table 1). In 2 studies investigating manifestations of chikungunya in patients requiring intensive care, a neurological disorder was the primary issue in 61%^32^ and 79%^33^ of chikungunya‐infected patients.

**Table 1:** List of neurological syndromes and syndromes and diseases associated with chikungunya virus

Described More Frequently	Described Less Frequently
Encephalopathy and encephalitis	Seizures with or without fever
Myelopathy and myelitis	Behavioural changes
Encephalomyelopathy	Sensorineural hearing loss
Myeloneuropathy	Stroke
Encephalomyeloneuropathy	Cerebellitis
Guillain‐Barré syndrome	Meningism
Acute disseminated encephalomyelitis	Third nerve palsy
Neonatal hypotonia	Encephaloneuropathy
Neuro‐ocular disease (uveitis, retinitis, optic neuritis)	Carpal tunnel syndrome
	Bilateral total ophthalmoplegia
	Mild encephalitis with a reversible lesion of the splenium
	Bickerstaff brainstem encephalitis–Miller Fisher syndrome–Guillain‐Barré syndrome overlap

In chikungunya‐associated neurological disease, the clinician must be vigilant for other complications in the same patient, a pattern also described in dengue but rarely in Zika infection. Amongst 99 cases of chikungunya‐associated neurological disease described in a study in India, 69 also had other complications involving, for example, the renal, hepatic, and respiratory systems.^30^ These patients should be managed using a multidisciplinary approach.

## 7 NEUROLOGICAL MANIFESTATIONS

**Figure 2:**
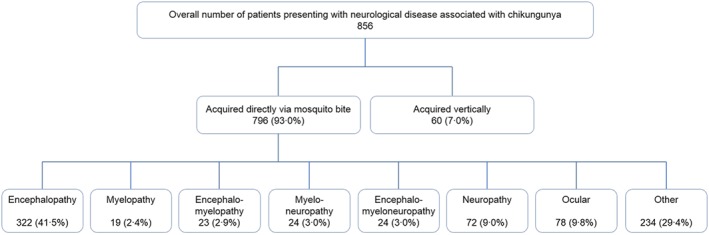
Presentations of nervous system disease associated with chikungunya infection

The literature on neurological manifestations of chikungunya reflects that of the disease activity itself, with 5 publications between 1964 and 1971 and 89 since 2005, comprising 27 case reports, 48 case series, 1 case‐control study, 3 cohort studies, 1 cross‐sectional study, and 14 pathogenesis studies (Figure 1); autopsy data were included in one report found.^34^ Table 2 provides a summary of all cases of reported chikungunya‐associated neurological disease. In total, we found 856 cases of chikungunya‐associated neurological disease in the literature; 796 (93.0%) were in adults and children infected directly via mosquito, 60 (7.0%) were in neonates infected vertically from mother to child (Figure 2). Because patients were investigated to variable extents, we have categorised them according to the presenting clinical syndromes of encephalopathy, myelopathy, neuropathy, combinations of these, and neuro‐ocular disease; we then provided diagnoses, treatment, and outcome where available. A limitation of our study was that we could not incorporate diagnostic criteria, given the range of information available amongst all the cases published. The most common clinical presentation of neurological disease associated with adult and child chikungunya infection was encephalopathy; it accounts for 322 (40.5%) of the 781 patients described. Excluding ocular disease, 474 (77.3%) of all adult and child cases had a pure CNS disorder, 82 (13.4%) had a pure peripheral nervous system disorder, and 57 (9.3%) had a disorder of both the CNS and peripheral nervous system. Cases in which coinfection of chikungunya with another neurovirulent arbovirus was detected are not included in Table 2 but are further discussed below.

**Table 2:** Reports of neurological disease associated with chikungunya virus in adults and children

Year of Case(s), Location	No.	Laboratory Evidence for Chikungunya	PL	CSF	Neurological Features	Diagnosis	Treatment^a^ and outcome
**Encephalopathy (n = 322)**							
1963‐1964, India^35^	2	HI	…	…	Unknown	Encephalitis	1 died, 1 unknown
1962‐1964, Thailand,^13^	1	HI/isol ser	…	…	Unknown	Meningoencephalitis	Unknown
2005‐2006, Mayotte^36^	2	PCR/IgM CSF/ser	…	…	Unknown	Meningoencephalitis	Unknown
2005‐2006, Réunion^33^	14	PCR CSF (2/12), PCR serum (4/10), IgM CSF (11/13), IgM serum (12/13)	…	…	Headaches, seizures, focal neurology, altered GCS	Encephalopathy	4 died
2005‐2006, Réunion^29^	84	PCR/IgM CSF/ser/BL	…	…	Unknown	Encephalitis (69), meningoencephalitis (15)	6 died
2005‐2006, Réunion^37^	57^b^	PCR CSF (40/52), PCR serum (31/37), IgM CSF (21/52), IgM serum (32/37)	…	WCC↑ (21/57), prot↑ (37/55)	International Encephalitis Consortium criteria used to classify patients	Encephalitis (24), nonencephalitic CHIKV‐associated CNS disease (33)	7 died, 12 disabled, 16 recovered, 22 unknown
2006, India^38^	11	IgM CSF/ser	…	…	Headaches, altered sensorium, ataxia, rigidity, opsoclonus; abnormal brain MRI (?no.)	Encephalopathy	3 died
2006, India^30^	37	Isol/PCR/IgM CSF/ser^c^	…	…	Unknown	Encephalitis	7 died
2006, India^39^	11	Isol/PCR/IgM/HI CSF/ser (6)	…	…	Unknown	Encephalitis	2 died
2006, Réunion^40^	16	PCR/IgM CSF/ser	…	…	Drowsiness, seizures, focal neurological signs; abnormal MRI brain (5) and EEG (?no.)	Encephalitis (12), encephalopathy (4)	2 died, 5 disabled, 9 no neurological sequelae
2006, India^41^	27	PCR CSF (4)	‖	WCC↑ (6/20), prot↑ (14/20)	59% abnormal behaviour; 22% drowsiness, extrapyramidal; 11% seizures; abnormal MRI brain (1/4)	Encephalitis	21 improved, 4 no improvement, 2 died
2007, Italy^42^	1	PCR CSF and ser, HI	5d	WCC↑, prot↑	83 y M; confusion, drowsiness	Encephalitis	Died after 3 d
2008, Singapore^43^	1	IgM ser	3d	…	45 y M; drowsiness, headache; abnormal MRI brain	Encephalitis	*Antimicrobials*. Full recovery
2009, Thailand^44^	2	IgM CSF	3 d	WCC↑, prot↑	27 y F; drowsiness; abnormal MRI brain	Meningoencephalitis	*Aciclovir*. Full recovery at 6 mo
		HI	0 d	85 y M; drowsiness, jerky movements; abnormal MRI brain	No improvement
2010‐2011, India^45^	4	PCR and IgM CSF, IgM ser	4 d	WCC↑, prot↑	32 y F; seizure, disorientation, neck stiffness	Encephalitis	Improved over 10 d
		PCR CSF and ser	…	WCC↑, prot↑	50 y M; headache, disorientation, drowsiness, meningism	Encephalitis	Improved over 7 d
		PCR CSF and IgM ser	3 d	WCC↑, prot↑	23 y M; seizure, dysarthria, hiccups, quadriparesis, CN involvement; abnormal MRI brain	Meningoencephalopathy	*MP*. Improved over 3 wk, mild weakness
		IgM CSF & ser	7 d	Prot↑	29 y F; headache, neck stiffness, quadriparesis, drowsiness; abnormal MRI brain and spine	ADEM	*MP*. Recovered at 1 mo, some residual weakness
2011,^d^ India^46^	1	IgM CSF and ser	10 d	NAD	55 y M; weakness, vertigo, ↓GCS, nystagmus, bulbar weakness; abnormal MRI brain and spine	Brainstem encephalitis (ADEM)	*MP*. Near‐complete recovery
2011‐2012, Cambodia^47^	11	PCR/isol CSF	…	…	<16 y; unknown	Meningoencephalitis	Unknown
2012,^d^ India^48^	1	IgM ser	7 d	WCC↑, prot N	32 y M; seizures, stimulus sensitive myoclonus	Meningoencephalitis	*Anticonvulsants*. Fully recovered at 3 mo
2014,^d^ India^49^	1	PCR, IgM CSF, and ser	2 d	…	12 y M; seizure, vomiting, altered sensorium, weakness, ↑UL tone and tremors, unequal pupils; abnormal CT	Encephalitis	Died after 6 d
2014, Tonga^50^	1	PCR ser, IgM CSF, and ser	7 d	WCC N, prot↑	57 y M; altered mental status, seizure; abnormal MRI brain and EEG	Encephalitis	*IVIG*, *anticonvulsants*. Improved
2014, L Antilles^32^	3	PCR/IgM CSF/ser	…	…	Met Venkatesan^51^ criteria for encephalitis	Encephalitis	Unknown
2015, Honduras^52^	18	PCR ser (11)^e^	…	WCC↑, prot↑	<12 mo (11); seizures/lethargy/bulging fontanelle/irritability/hyperalgesia; abnormal MRI brain (5/5), abnormal EEG (7/14)	Meningoencephalitis	1 died, remaining unknown
2015,^d^ India^53^	3	IgM CSF/ser	…	NAD	19 d F; tonic seizures, poor feeding	Encephalopathy	Unknown
				WCC↑, prot N	23 d M; multifocal clonic seizures	Encephalopathy	
				NAD	25 d F; multifocal clonic seizures, poor feeding
2015,^d^ Colombia^54^	1	IgM CSF/ser	4 d	WCC↑,^f^ prot↑	23 d M; seizures, stupor, severe thrombocytopenia; abnormal MRI brain	Encephalitis	↓hearing, ↓tone, motor delays at 13 mo
2016,^d^ India^55^	1	PCR CSF and ser	…	NAD	55 y M; altered sensorium, GCS 12; abnormal MRI brain	Mild encephalitis with a reversible lesion of the splenium	Full recovery at 5 d
2016, Brazil^g^	2	PCR CSF and ser, IgM ser	6 d	WCC↑, prot N	51y F; confusion, seizure, drowsiness, dysarthria	Encephalitis	*Aciclovir*. Full recovery
		PCR CSF	4 d	WCC↑, prot↑	84y M; confusion, dysarthria, dysphagia, quadriparesis; NP: myositis		*IVIG*, *aciclovir*, *antibiotics*, *antifungals*. No improvement
2016, India^56^	3	PCR ser	…	…	Children; seizures/altered sensorium	Meningoencephalitis	Recovered at 3‐4 d (2), died after 6 h (1)
2016,^d^ Brazil^57^	2	IgM CSF/ser	4d	WCC↑, prot↑	74 y M; confusion, drowsiness, paraparesis; abnormal MRI brain; NP: AMSAN	Encephalitis	*IVIG*, *plasmapheresis*. Improved at 3 mo
			6d		83 y M; confusion, lethargy, required ventilation		*Aciclovir*, *IVIG*. Complete recovery at 8 d
2016, Brazil^58^	3	IgM ser	1 d	…	<5 y M; headache, seizures, GCS 3, areflexia; abnormal CT brain	Not given	Died 23 d after admission
			10 d	WCC N, prot↑	65 y M; seizures, GCS 9, required ventilation; abnormal CT brain		*Aciclovir*. Died 1 d after admission
			9 d	…	92 y F; ↓GCS, LL involuntary movements, required ventilation		Died 10 d after admission
2017,^d^ Brazil^59^	1	PCR CSF and ser and urine and saliva	13 d	WCC↑, prot↑	57 y M; confusion	Meningoencephalitis, anterior uveitis^h^	*Aciclovir, steroids po and top*, *tropicamide top*. Improved
**Myelopathy (n = 19)**							
2006, India^38^	4	IgM CSF/ser	…	WCC↑, prot↑	UR followed by paraparesis	Myelopathy	Improvement (unclear extent)
2006, India^39^	5	Isol/PCR/IgM/HI CSF/ser (2)	…	…	Unknown	Myelitis	Unknown
2006, India^41^	7	PCR CSF (2), isol (1)	—i	WCC↑ (2/6), prot↑ (2/6)	Para/quadriparesis (7), UR (6); abnormal MRI spine (1)	Myelopathy	5 improved, 2 no improvement
2015,^d^ India^60^	1	IgM CSF/ser	2 wk	WCC N, prot↑	18 y M; quadriparesis, ↓sensation, UR, areflexia, myositis (↑creatine kinase); abnormal MRI C1‐C6	Myelitis	*MP*. Slow, partial improvement
2016, Brazil^61^	1	IgM ser	8 d	…	Paraparesis, T10 sensory level	Myelitis	*Steroids po*. Fully recovered at 2 mo
2016, Brazil^g^	1	PCR CSF	0 d	NAD	20 y M; paraesthesia, triparesis, hyperreflexia, C6 sensory level, UR; abnormal MRI spine; NP NAD	Myelitis	*MP*. Partially improved
**Encephalomyelopathy (n = 23)**							
2005‐2006, Réunion^29^	1	PCR/IgM CSF/ser/BL	…	…	Unknown	Myelomeningoencephalitis	Unknown
2006, India^38^	7	IgM CSF/ser	…	…	Unknown	Encephalomyelopathy	Unknown
2006, India^30^	14	Isol/PCR/IgM CSF/ser^c^	…	…	Unknown	Encephalomyelitis (11), encephalomyelopathy (3)	5 died
2016, Brazil^g^	1	PCR ser and urine, IgM CSF and ser	0 d	WCC↑, prot↑	76 y M; seizures, confusion, dysarthria, headache, neck stiffness, spastic paraparesis, T2‐T3 sensory level, UI	Encephalomyelitis	*Antivirals*, *antibiotics*. Unknown
**Myeloneuropathy (n = 24)**							
2006, India^38^	13	IgM CSF/ser			Unknown	Myeloneuropathy	Unknown
2006, India^41^	7	PCR CSF (1)	<5 to 10‐20 d	WCC N (6/6), prot↑ (5/6)	Quadriparesis (6), UR (1); abnormal MRI spine (3); NP AIDP (7)	Myeloneuropathy	4 improved, 2 no improvement, 1 died
2009, Thailand^44^	1	IgM CSF	2 wk	WCC N, prot↑	44 y F; quadriparesis, dysphonia/phagia, facial diplegia, areflexia; abnormal MRI C4‐C5; NP AMSAN	Myeloneuropathy	*IVIG*. Rapid improvement, fully recovered at 6 mo
2012,^d^ India^62^	1	IgM ser	20 d	WCC↑, prot↑	56 y M; weakness and sensory loss; abnormal MRI spine C2‐C3 T5‐T7	Myeloradiculopathy	Improved
2014, Dom Rep^63^	1	IgM and IgG ser	10 d	WCC↑, prot↑	47 y F; L LL weakness, R LL pain, T12 sensory level; abnormal MRI T12‐L1 + cauda equina	Myeloradiculopathy	*MP*. Recovered at 6 mo, residual pain
2016, Brazil^g^	1	PCR and IgM CSF	2 d	WCC N, prot↑	63 y M; paraesthesia; flaccid areflexic paraparesis; T4 sensory level; UR; NP AMSAN	Myeloradiculitis	*IVIG*. No improvement
**Encephalomyeloneuropathy (n = 24)**							
2006, India^38^	9	IgM CSF/ser	…	…	Unknown	Encephalomyeloneuropathy	Unknown
2006, India^30^	12	Isol/PCR/IgM CSF/ser	…	…	Unknown	Encephalomyeloneuritis (9), encephalomyeloneuropathy (3)	1 died
2008,^d^ India^34^	2	IgM CSF and ser	…	WCC↑,^j^ prot↑	65 y M; drowsiness, neck stiffness, weakness; abnormal MRI brain and nerve root; NP AMAN	Encephalomyeloradiculitis	*MP*. No improvement
				WCC↑, prot↑	74 y M; drowsiness, weakness; abnormal MRI brain and nerve root; NP “generalised sensorimotor peripheral neuropathy”		*Dexamethasone*. Died; brain autopsy gross oedema, cerebellar haemorrhages, small foci demyelination
2009, Singapore^64^	1	PCR CSF, ser, urine, skin	2 d	WCC N, prot↑	54 y M; weakness, confusion, vomiting, sensory level, shock, rhabdomyolysis, UR; NP AIDP; EEG encephalopathy	AIDP	*IVIG*. Full recovery
**Neuropathy (n = 72)**							
1963‐1964, India^65^	1	HI ser	…	WCC↑,^k^ prot↑	Quadriparesis, facial diplegia, ↓R visual acuity	GBS	Complete slow recovery over months
2005‐2006, Réunion^29^	4	PCR/IgM CSF/ser/BL	…	…	Unknown	GBS	Unknown
2005‐2006, Réunion^33^	1	IgM CSF and ser	…	WCC N, prot↑	55 y M; weakness, hyporeflexia, facial palsy; NP “suggestive” of GBS	GBS	Moderately disabled at 6 mo
2005‐2006, Réunion^66^	2	IgM CSF	…	WCC N, prot↑	NP sensory motor deficit (2)	GBS	*IVIG*. Rapid improvement
2006, India^41^	7	PCR CSF (1)	—i	Prot↑(5/6), WCC N (6/6)	Quadriparesis with AIDP (7)	Peripheral neuropathy	6 improved; 1 no improvement
2006, Réunion^67^	3	IgM ser	2 wk	WCC N, prot↑	51y F; quadriparesis, areflexia, facial diplegia; NP AIDP	GBS	*IVIG*. Partial recovery at 1 mo
		PCR ser	3 d	NAD	60 y M; quadriparesis, L facial palsy, hypoaesthesia, areflexia; NP AIDP		*IVIG*. Good recovery at 1 mo, residual palsy
		IgM & IgG ser	1 wk	WCC N, prot↑	49 y F; paraparesis, proprioceptive ataxia, areflexia, facial diplegia, dyspnoea; NP AIDP		*IVIG*. Good recovery at 1 mo, residual palsy
2006, India^38^	13	IgM CSF/ser	…	…	Unknown	Neuropathy	Unknown
2006, Réunion^68^	2	IgM CSF & ser	1 wk	WCC N, prot↑	51 y F; areflexia, facial diplegia, dyspnoea requiring ventilation; NP AIDP	GBS	*IVIG*. Good recovery at 2 mo
		IgM ser	2 wk		48 y F; weakness, paraesthesia, areflexia, dyspnoea; NP “peripheral neuropathy, conduction block”		*IVIG*. Good recovery
2006, India^39^	2	Nil	…	…	Unknown	GBS	Unknown
2006, India^l^,^69^	4	IgM CSF/ser	…	…	Progressive, symmetrical, ascending quadriparesis with areflexia (4), required ventilation (1)	Acute flaccid paralysis	*MP*. Improved: rapidly (3), by 1 mo (1)
2014, L Antilles^32^	6	PCR/IgM CSF/ser	…	…	Unknown	GBS	Unknown
2014‐2015, Fr Poly^70^	9	PCR/IgM CSF/ser	…	WCC N, prot↑	Sensorimotor deficit (8), facial diplegia (1); NP mixed axonal and demyelinating (9)	GBS	*IVIG* (9). NP returned to approximately normal at 3 mo
2014‐2015, L Antilles^71^	13	PCR CSF (3)/ser (3), IgM ser (13)	1‐22 d	WCC N, prot↑	Mean age 61; severe (6), autonomic dysfunction (5), required ventilation (5)	AIDP (7), AMSAN (2), MFS (2), pharyngeal‐cervical‐brachial weakness (1), Bickerstaff brainstem encephalitis (1)	*IVIG* (12), *plasma exchange* (2). 2 died, 8 improved, 1 severe residual symptoms, 1 CIDP, 1 unknown
2016, Brazil^g^	1	PCR urine, IgM ser	7 d	WCC↑, prot↑	67 y F; flaccid areflexic quadriparesis, dysphagia, impaired sensation; NP AMSAN	GBS	*IVIG*. No improvement
2016,^d^ Colombia^72^	1	PCR and IgM ser	…	WCC N, prot↑	77 y F; paraesthesia, bilateral hemiparesis, impaired sensation, hyporeflexia; NP AIDP	GBS	*IVIG*. Fully recovered at 8 wk
2016, Brazil^58^	1	IgM ser	15 d	WCC↑, prot↑	51 y F; quadriparesis, neck stiffness	Not given	*Antibiotics*. Died after 38 h
2016,^d^ India^73^	2	IgM CSF/ser	17 d	…	18 y M; areflexic quadriparesis, dysphagia, dyspnoea; NP AMAN	GBS	*Plasmapheresis*. Partial improvement
			12 d	…	20 y M; flaccid quadriparesis, facial and bulbar weakness; NP AIDP		
**Ocular disease (n = 78)** h							
2006, India^74^	14	IgM ser	11.0 d^m^	…	Visual field defect (14), ↓visual acuity (13), pain (1), floaters (1), diplopia (1), RAPD (9), disc oedema (9), VII CN palsy (2), delayed VEP	Papillitis (6), neuroretinitis (3), retrobulbar neuritis (3), demyelination optic tract (2)	*MP 3 d*, *steroids po 2 wk*. 10 improved, 4 poor outcome
2006, India^75^	37	IgM ser	33.2 d^m^	…	Primary presenting complaint ↓visual acuity, unilateral (30), bilateral (7)	Anterior uveitis (11), panuveitis (5), optic neuritis (4), lagophthalmos and sixth nerve palsy (3), retrobulbar neuritis (3), retinitis and vitritis (2), bilateral neuroretinitis (1), keratitis (3), CRAO (1), choroiditis (2), retinal detachment (2)	Visual acuity of 26 followed up: 11 improved, 12 remained stable, 3 worsened
2006, India^38^	2	IgM CSF/ser	…	…	↓visual acuity	Bilateral retinal haemorrhage (1), branch retinal artery occlusion (1)	*Steroids intravitreal*. Minimal (1)/partial (1) improvement
2006, India^76^	9	IgM ser	4‐12 wk	…	↓visual acuity/pain/red eye	Episcleritis (1), anterior uveitis (5), retinitis (3)	*Indomethacin po*; *steroids*, *homatropine*, *diclofenac*, *timolol top*; *aciclovir iv/po*, *steroids po*. Recovered well (9)
2007, India^77^	10	IgM ser	1‐6 wk	…	↓visual acuity (10), pain (10), bilateral (3), visual field defects (10), disc oedema (10), RAPD (7), delayed VEP	Papillitis (7), retrobulbar neuritis (1), perineuritis (1), neuroretinitis (1)	*MP 3 d*, *steroids po 2 wk*. Rapid improvement (9), persistent RAPD/visual field/colour vision defect (4/6/2)
2007,^d^ India^78^	1	PCR and IgM ser	2 wk	…	48 y F; bilateral ↓visual acuity, bilateral centrocaecal scotoma, retinal haemorrhage	Bilateral neuroretinitis	*Steroids po*. Visual acuity 20/30 R, 20/20 L at 2 mo
2009,^d^ India^79^	1	PCR aqueous humour	1 wk	…	20 y F; L ↓visual acuity, tripod dendritic pattern of keratic precipitates	Fuchs heterochromic iridocyclitis and cataract	*Cataract surgery*. Recovered well
2010, India^80^	1	IgM ser	4 wk	…	27 y F; bilateral ↓visual acuity, R RAPD, bilateral retinitis posterior pole, macular oedema, serous detachment	Anterior uveitis and retinitis	*Steroids po*. Gradual recovery
2011,^d^ India^81^	1	PCR ser	1 wk	…	65 y M; bilateral ↓visual acuity, neuroretinitis, cotton wool spots, retinal haemorrhages	Bilateral neuroretinitis	*Steroids, aciclovir po*. Partial improvement
2015,^d^ Dom Rep^82^	1	IgM and IgG ser	4 d	NAD	47 y F; bilateral ↓visual acuity, photophobia, optic nerve head oedema, hyperpigmented scars, serous detachment temporal macula, represented with floaters; abnormal MRI orbits	Panuveitis, retinal detachment	*Steroids po and top*, *cyclopentolate top*, *mycophenolate*. Improved
2016,^d^ Dom Rep^83^	1	IgM and IgG ser	20 d	…	44 y F; ↓visual acuity, floaters, keratic precipitates, anterior chamber cells, Koeppe nodules	Intermediate uveitis	*Steroids po and top*. Rapid improvement
**Other focal neurology (n = 233)**							
1962‐1964, Thailand^13^	1	HI/isol CSF/ser	…	…	Febrile convulsions	Febrile convulsions	Unknown
1963‐1964, India^65^	2	HI	14 d	…	Limb paresis and slurring of speech	Not given	Recovered
			7 d		Vocal hoarseness and nasal regurgitation		
1964, India^65^	1	HI/isol CSF/ser	4 d	…	12 y M; bilateral total ophthalmoplegia, loss of accommodation reflex	CN palsy	Complete recovery at 1 wk
	3	HI/isol CSF/ser	…	NAD	Delirium, coma, meningism, sluggish pupils, dysarthria	Not given	Unknown
1964, India^84^, ^85^	12	PCR/IgM CSF/ser	…	…	Seizures in infants (3), children (9); associated with fever (12), focal (2), ↓GCS (4)	Seizures	Died (1), residual neurological deficit (2)
2005‐2006, Réunion^66^	21	PCR/IgM CSF/ser	…	WCC N, prot↑ (12)	Confusion (20), headache (7), epilepsy (6), meningism (1), motor deficit (1), sensory deficit (2); EEG diffuse slowing (13), epileptic activity (3), NAD (2)	Not given	Died (5), generally good outcome (16)
2005‐2006, Réunion^29^	12	PCR/IgM CSF/ser/BL	…	…	Seizures	Seizures	Unknown
2005‐206, Réunion^29^	5	PCR/IgM CSF/ser/BL	…	…	Unknown	Stroke (2), cerebellitis (3)	Unknown
2006, Réunion^40^	14	PCR/IgM CSF/ser	…	…	Nuchal rigidity, Kernig/Brudzinski sign, photophobia, tense fontanelle (4)	Meningeal syndrome	Mild/no neurological sequelae (1)/(3)
						Febrile seizures (10)	Asthenia (1), no neurological sequelae (9)
2006, India^86^	20	IgM ser	…	WCC↑ (9), prot↑ (20)	Altered mental status (20), psychosis (6), seizures (15), CN deficit (20), hemiparesis (1), LMN paraparesis (3), involuntary movements (4), optic neuritis (2)	Not clear	13 gradual full improvement, 1 blind, 6 died
2006, India^87^	8	PCR CSF/ser	…	WCC↑ (2/5)	Altered mental status, meningism, seizures, status epilepticus, aphasia	Not given	At discharge, normal GCS (6), ↓GCS (2)
2006, Réunion^88^	25	PCR CSF (8), IgM/PCR ser	…	WCC↑ (1/17), prot↑ (3/17)	Paediatric cohort: convulsion, confusion, behavioural disorders, meningism; abnormal MRI (2/8), abnormal EEG (8/10)	Not given	Unknown
2006, India^38^	18	IgM CSF/ser	…	…	Unknown	Encephaloneuropathy (8), carpal tunnel syndrome (10)	Unknown
2007,^d^ India^89^	1	IgM CSF/ser	13 d	WCC N, prot↑	45 y M; asymmetric quadriparesis, dysphagia, clonus, dystonia; abnormal MRI brain	ADEM	*MP*. Walking independently at 12 d
2007,^d^ India^90^	1	IgM ser	2‐3 d	…	15 y F; sudden‐onset profound L‐sided hearing loss, tinnitus	Sensorineural hearing loss	No improvement at 1 mo
2011,^d^ India^91^	1	IgM ser	5 d	WCC↑, prot↑	26 y F; spastic quadriplegia, impaired sensation, UR; abnormal MRI brain and spine	ADEM	*MP*. Good clinical and radiological recovery
2013,^d^ India^92^	1	IgM CSF/ser	9 d	WCC↑, prot↑	8 y M; flaccid quadriparesis, R UMN facial palsy, seizure, UR; abnormal MRI brain and spine	ADEM	*MP*. Minimal improvement at 6 mo
2014, Martinique^93^	1	IgM and IgG ser	2 d	…	62 y M; isolated unilateral third nerve palsy	CN palsy	Improved at 6 mo
2014, L Antilles^32^	2	PCR/IgM CSF/ser	…	…	Diffuse brain ischaemia leading to brain death	Not given	Died
2014, Fr Poly^7^	1	IgM ser	6 d	WCC↑, prot↑	74 y M; hypoesthesia, flaccid quadriplegia, dyspnoea, GCS 3, CN palsies, required ventilation; abnormal MRI brain, abnormal EEG, NP axonal polyneuropathy	Bickerstaff brainstem encephalitis‐MFS‐GBS overlap	*IVIG*, *MP*. Normal mental function and residual paresis at 1.5 y
2015, Honduras^52^	59	PCR ser	…	…	Seizures	Seizures	Unknown
2016, India^56^	2	PCR ser (1), IgM ser (1)	<7 d	…	Children; hyperactivity, insomnia, aggressive behaviour, hallucinations, behaviour changes	Not given	Recovered at 4 d (1), persistent behavioural problems at 4 wk (1)
2016, Brazil^61^	21	IgM ser	…	…	Seizures, altered consciousness, weakness, impaired sensation, sphincter dysfunction, persecutory delusions, suicidal/aggressive behaviour, insomnia, headache	Not given	Unknown
2016, Brazil^g^	1	PCR ser and urine, IgM ser	16d	Prot N	17 y M; L hemiparesis and numbness, facial palsy, impaired sensation; abnormal MRI brain	ADEM	*MP*. Improved
2017,^d^ India^94^	1	IgM ser	10d	…	5 y F; bilateral ophthalmoplegia, blurring of vision	Bilateral ophthalmoplegia	*Steroids po*. Unknown

Abbreviations: /, or (eg, IgM CSF/ser = not specified whether IgM detected in CSF or serum); …, data unavailable; ADEM, acute disseminated encephalomyelitis; AIDP, acute inflammatory demyelinating polyneuropathy; AMAN, acute motor axonal neuropathy; AMSAN, acute motor and sensory axonal neuropathy; BL, bullous lesions; CHIKV, chikungunya virus; CIDP, chronic inflammatory demyelinating polyneuropathy; CN, cranial nerve; CRAO, central retinal artery occlusion; CSF, cerebrospinal fluid; CT, computed tomography; Dom Rep, Dominican Republic; EEG, electroencephalogram; F, female; Fr Poly, French Polynesia; GBS, Guillain‐Barré syndrome; GCS, Glasgow coma score; HI, haemagglutination inhibition; IgM, immunoglobulin M; IVIG, intravenous immunoglobulin; isol = viral isolation; L, left; L Antilles, Lesser Antilles; LL, lower limb; LMN, lower motor neuron; M, male; MFS, Miller Fisher syndrome; MP, methylprednisolone; MRI, magnetic resonance imaging; N, normal; NAD, no abnormality detected; PCR, polymerase chain reaction; PL, prodrome length (time between initial infection and onset of neurology); prot, protein (↑, >0.4 g/L for adults, >1.5 g/L for neonates); R, right; RAPD, relative afferent papillary defect; ser, serum; UI, urinary incontinence; UL, upper limb; UMN, upper motor neuron; UR, urinary retention; VEP, visual evoked potential; WCC, white cell count (↑, >5 cells/μL).

a Treatments are in italics.

b Five vertically transmitted cases.

c Neurological sequelae in cases with positive dengue virus IgM as well as CHIKV were attributed to CHIKV.

d Date of article submission, date of case unclear.

e Seven of these patients did not have laboratory evidence of chikungunya infection and were not described further; their age and whether they were vertically transmitted cases were not reported.

f WCC reported as “>5”; normal neonatal WCC ranges from 0 to 30 cells/μL.

g Unpublished findings from Mehta R, Soares C, Medialdea‐Carrera R, et?al. (2017) “The spectrum of neurological disease associated with Zika and chikungunya viruses in adults in Rio de Janeiro, Brazil: a case series.”

h One patient with both encephalitis and anterior uveitis has not been included in the ocular disease section.

i Range of prodrome lengths: <5 to 10‐20 d (24) and >30 d (3) for encephalitis; <5 to 20‐30 d for myelopathy; <5 to 10‐20 d for GBS.

j WCC reported as “few cells”; unclear actual number per microlitre.

k WCC mildly elevated, 8 lymphocytes/field.

l Andaman and Nicobar Islands.

m Mean.

**Figure 3:**
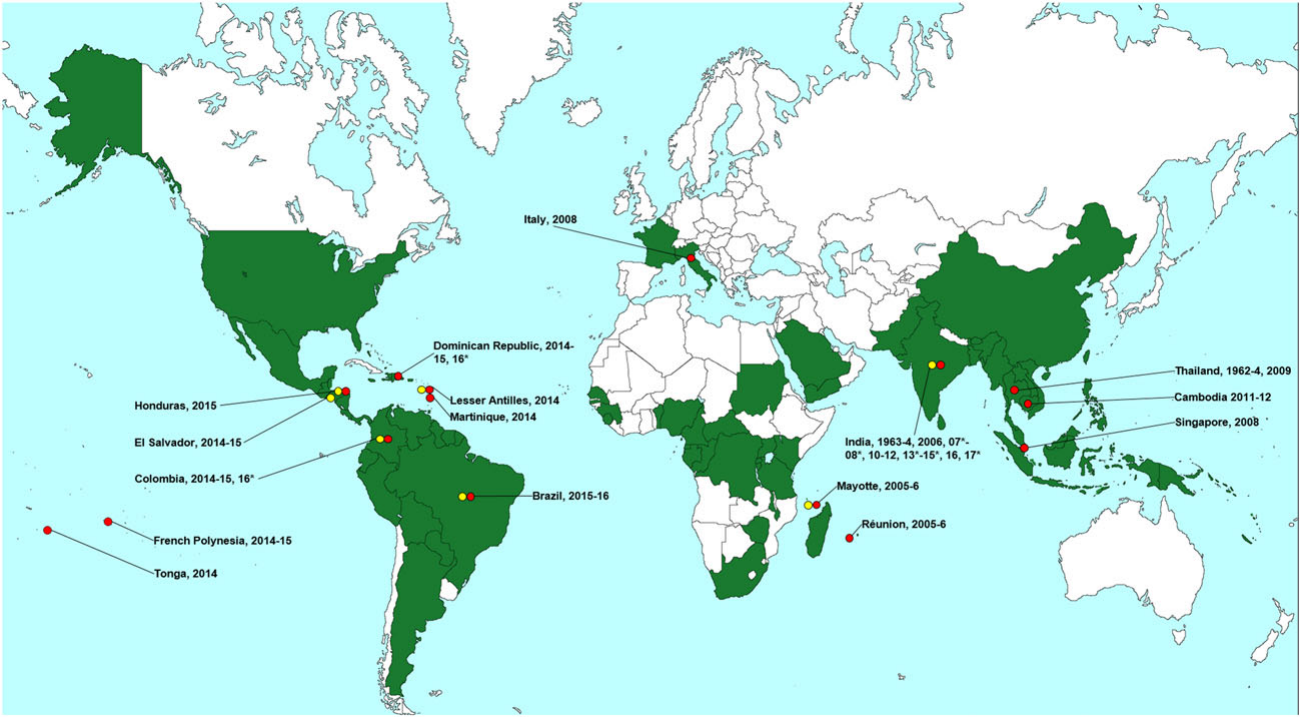
Global distribution of chikungunya virus and countries/territories with reported associated neurology. Key: 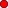 adult and child neurological disease associated with chikungunya infection; 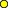 vertically acquired neurological disease in the neonate associated with chikungunya infection. *unclear date of case(s), year refers to year of publication. Data regarding global distribution of chikungunya virus acquired from the Centers for Disease Control and Prevention website^95^

Neurological disease following chikungunya virus infection was first reported during an outbreak in 1964 in Madras, India.^65^ Four cases with chikungunya confirmed serologically or by viral isolation were described. Two presented with a meningoencephalitic picture (“delirium or coma, and signs of meningeal irritation with nuchal rigidity and Kernig's sign, sluggish pupillary reaction etc”), one with acute flaccid paralysis and elevated CSF protein, suggestive of Guillain‐Barré syndrome (GBS), and one with transient dysarthria. Since then, neurological manifestations have been reported throughout the Indian Ocean, South Asia, the Pacific islands, Southern Europe, the Caribbean, and South America, ranging from mild behavioural disorders to severe acute syndromes of both CNS and peripheral nervous system (Figure 3).

Given the large spectrum of neurological disease and scarce epidemiological data, estimating the incidence of neurological disease amongst all systemically symptomatic chikungunya infections is difficult. In one study from the 2006 Indian outbreak,^39^ 18 (4.4%) of 405 suspected chikungunya cases attending the recruiting hospital over 3 months developed neurological complications; this study did not, of course, include the many people with uncomplicated chikungunya infection who did not seek hospital attention. An epidemiological study of the 2005 to 2006 Réunion Island outbreak found approximately 0.3% of all chikungunya infections resulted in “atypical” cases,^29^ of which 24.1% of the adults presented with abnormal neurology. Thus, approximately 0.1% (1 case per 1000) of all chikungunya infections developed neurological disease.

It has been suggested that severe complications of chikungunya infection typically arise in those with co‐morbidities.^96^ The above epidemiological study describing 610 atypical cases of chikungunya infection showed that underlying respiratory disease, cardiac disease, and hypertension were all independently associated with severe complications, including neurological disease.^29^ However, a study from India of 124 atypical chikungunya cases did not identify co‐morbidity as a significant risk factor for systemic complications or fatality^30^; similarly, in a case series of chikungunya‐associated GBS, 6 (67%) of 9 cases did not have any co‐morbidities.^70^ Thus, although underlying co‐morbidities may play a role in neurological and other complications of chikungunya, they are not an indispensable requisite. Interestingly, age has been consistently reported as a significant risk factor for severe manifestations of chikungunya infection, both in the elderly (>60‐65)^29^, ^30^, ^37^ and in infants.^37^

Future studies will need to determine whether initially asymptomatic chikungunya infections, ie, without a primary fever, arthralgia, or rash syndrome, can cause neurological disease. Although none have been reported to date, the possibility has not yet been investigated in adults. In vertical transmission, a retrospective study from the Réunion Island outbreak found that of 38 symptomatic neonates (with combinations of fever, rash, and oedema), 2 mothers had been asymptomatic (with a positive chikungunya PCR or IgM).^97^ However, mild symptoms may have been missed by the mothers owing to poor recall, and whether their neonates developed neurological manifestations was unclear.

Cocirculation of chikungunya, Zika, and dengue viruses has been reported in much of South America^6^ and is a potential problem in all areas of the world where *Aedes* mosquitoes are endemic. Aedes albopictus mosquitoes have the ability to deliver more than 1 arbovirus in their saliva, raising the possibility of simultaneous transmission of the viruses.^98^ Furthermore, coinfection of arboviruses has been detected in patients presenting with neurological disease.^99^, ^100^ Given that all 3 arboviruses are known to be neurovirulent, it is unclear whether in these patients, their neurological disease is associated with one or more of their infections. Coinfection has also been reported in pregnant women,^101^, ^102^ the significance of which for the neonate is unclear. The most common coinfection to be reported in all patients is with chikungunya and dengue viruses, although this may be due to the greater number of epidemics of these viruses so far compared with Zika. Albeit rarely, coinfection with all 3 viruses has been reported, including in patients with neurological disease.^99^, ^103^ The differences in disease pathogenesis, presentation, and severity between monoinfections and coinfections are currently unknown. It is clear, however, that in endemic areas, pregnant patients with a fever‐arthralgia‐rash syndrome and all patients presenting with acute neurological disease (regardless of previous viral symptoms) should be investigated for all 3 of chikungunya, Zika, and dengue viruses.

The following sections evaluate the evidence for each of the neurological syndromes that has been described in association with chikungunya virus infection.

### 7.1 Encephalopathy and encephalitis

Encephalopathy, defined by the International Encephalitis Consortium as “a clinical state of altered mental status, manifesting as confusion, disorientation, behavioural changes or other cognitive impairment,”^51^ is one of the most common neurological presentations for arboviral infections. Whilst in some patients this may be due to encephalitis—ie, brain inflammation associated with direct viral infection—in others, it may be a non‐specific manifestation of a severe systemic disease, for example, due to hypoperfusion of the brain.^3^, ^5^, ^104^ Strictly speaking, encephalitis is a pathological diagnosis, but for practical purposes, it can be diagnosed in an encephalopathic patient if there is surrogate evidence of brain inflammation, for example, from a CSF pleocytosis, brain imaging, or focal changes on electroencephalogram.^2^, ^5^

In one study from Réunion Island, where the authors were careful to accurately define encephalitis using the international criteria, the estimated cumulative incidence rate for chikungunya‐associated encephalitis was 8.6 per 100 000 people during the 2005 to 2006 outbreak, which led to a 2‐fold increased incidence of all encephalitis in the region.^37^ Twenty‐four patients with encephalitis were reported (5 of whom were neonates infected perinatally), presenting with altered mental status, as well as seizures and focal neurological signs in some. When compared with encephalopathic patients who did not meet criteria for encephalitis, the encephalitic patients had more severe CNS disease and required more intensive care support; this was despite there being no significant difference between chikungunya viral loads and IgM titres in the serum or CSF, which may indicate a role for predisposing host factors in chikungunya virus neurovirulence. Overall, our literature review revealed that 251 of the 322 (78.0%) patients who presented with an isolated encephalopathy syndrome had a diagnosis of encephalitis, whereas 66 (20.5%) and 2 (0.6%) had a diagnosis of encephalopathy and acute disseminated encephalomyelitis, respectively. Additionally, involvement of the meninges was also reported in 55 (17.1%) cases.

**Figure 4:**
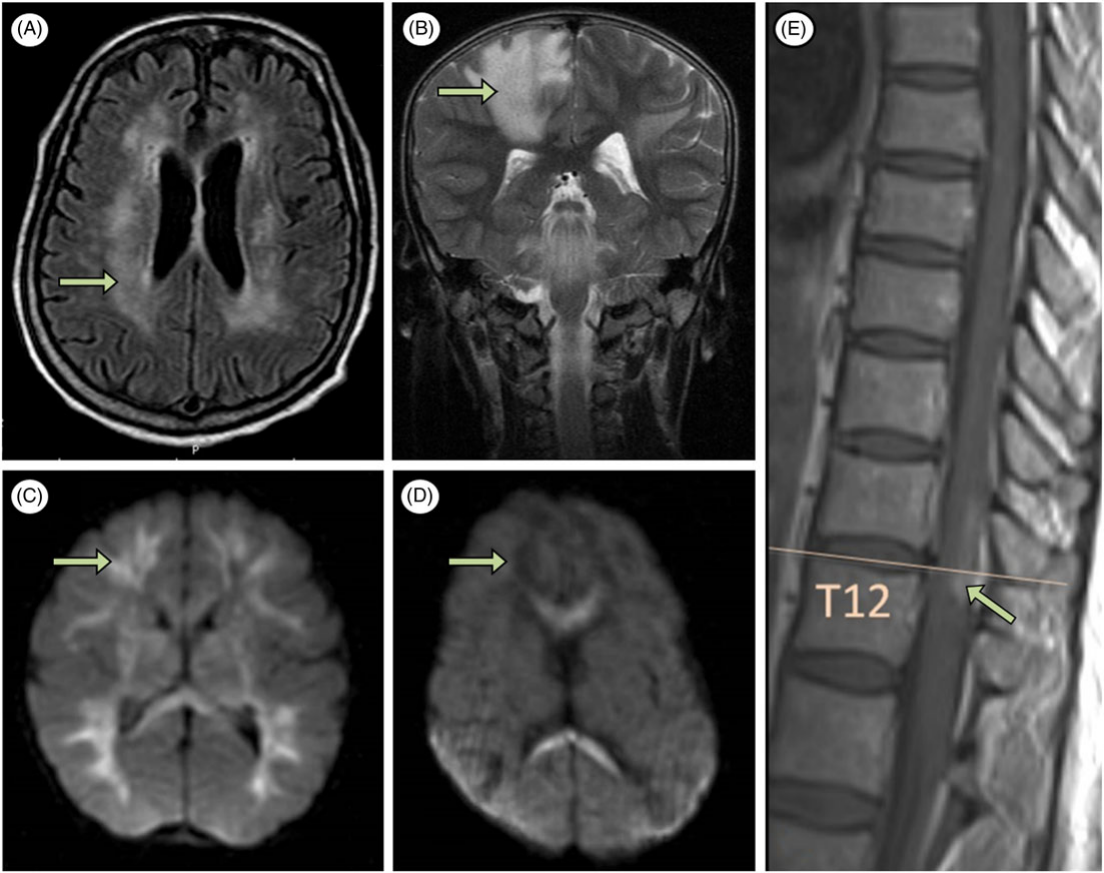
Central nervous system imaging abnormalities in patients with chikungunya infection. A, Signal abnormality involving the periventricular white matter in an 85‐year‐old patient with encephalitis (axial fluid‐attenuated inversion recovery).^44^ B, Confluent areas of signal abnormality consistent with demyelination in an 8‐year‐old patient with acute disseminated encephalomyelitis (coronal T2).^92^ C, Signal abnormality (hyperintense) involving the corpus callosum and the frontal and parietal lobes in neonate A with vertically acquired encephalopathy (day 6, axial diffusion weighted imaging).^105^ D, Signal abnormality (hypointense) involving the frontal and parietal lobes in neonate B with vertically acquired encephalopathy (day 21, axial diffusion‐weighted imaging).^105^ E, Signal abnormality at T12 in a 47‐year‐old patient with myeloradiculopathy (sagittal T1)^63^

Symptoms of encephalitis begin between 0 and 13 days following the onset of systemic features of infection (see Table 2). As is the case in other arboviral encephalitides, a CSF pleocytosis is not always seen.^40^, ^41^ Unlike encephalitis caused by other CNS pathogens such as herpes simplex virus and cytomegalovirus, which have characteristic imaging abnormalities, chikungunya encephalitis in adults and children does not appear to show a distinct pattern. Described abnormalities include oedema or non‐specific haemorrhage on computed tomography, and increased T2+/− fluid‐attenuated inversion recovery signal (Figure 4) or restricted diffusion signal in several areas of the cerebrum on magnetic resonance imaging (MRI).^34^, ^40^, ^44^ Many cases do not show any imaging abnormalities at all.^37^, ^40^ Although there are non‐specific slowing of brain waves in some patients, there is no specific electroencephalogram pattern.^40^, ^50^

In the Réunion Island study, 7 of the 57 patients (aged 4 d‐88 y) with either encephalopathy or encephalitis died in hospital or shortly after discharge; of the 10 adults followed up after 3 years, 3 had persistent neurological sequelae in the form of epilepsy, postinfectious dementia, and cognitive disorder.^37^ Taking into account the attrition in the follow‐up cohort, a range from 18% to 43% of patients were estimated to have neurological sequelae. Similarly, a paediatric series from the same outbreak reported 5 (31%) of 16 children with chikungunya‐associated encephalopathy or encephalitis had residual neurological deficit, whilst 2 (13%) died.^40^ From our literature review, of the 127 patients cases for whom follow‐up data were reported, 62 (48.8%) had complete or near‐complete recovery, 25 (19.7%) had residual neurological deficit, and 40 (31.5%) died.

### 7.2 Myelopathy and myelitis

Chikungunya virus can cause myelopathy, symptoms of spinal cord disease, which may present with limb weakness, sensory changes, hyperreflexia, and bowel and bladder disturbances, depending on the level of the lesion and extent to which the cord is involved. If cord inflammation is confirmed by MRI, a CSF pleocytosis, or elevated CSF IgG index, showing local immunoglobulin production, then it is classified as myelitis.^106^

The incidence of spinal cord disease after chikungunya infection is not known, but it is likely to be less than that of encephalopathy, given that the 90 cases described in the literature comprise less than a third of the 322 cases of encephalopathy. Myelopathy and myelitis usually occur as part of more widespread neurological disease. Of the 90 patients identified, 47 had myelopathy as part of more widespread CNS disease, with encephalopathy or encephalitis; for 48, there was also peripheral nervous system disease in the form or radiculopathy or neuropathy; just 19 patients had a pure myelopathy syndrome. Spinal cord disease typically presents 0 day (Mehta et al, unpublished findings) to 3 weeks after the first clinical feature of infection (fever, arthralgia, or rash).^41^ Patients present with weakness in 2, 3, or 4 limbs, sometimes accompanied by one or more of paraesthesia in the limbs, a sensory level, or urinary retention. In our literature review, of the 12 patients with a pure myelopathy where CSF data were provided, 6 (50%) had a CSF pleocytosis. Similarly, MRI abnormalities are variable. They may range from changes “suggestive of demyelinating pathology” to extensive T2/fluid‐attenuated inversion recovery hyperintensity from the cervicomedullary junction to the C6 level.^60^

No deaths have been reported for chikungunya‐infected patients with a pure myelopathy syndrome; follow‐up data were reported for 13 patients, 11 of whom improved, although the extent of this was often unclear.

### 7.3 Acute disseminated encephalomyelitis

Like other acute viral infections, chikungunya can trigger an acute inflammatory syndrome involving the brain parenchyma and spinal cord, which is thought to be an immune‐mediated response to infection, rather than due to direct viral invasion. The diagnosis of this monophasic illness is usually based on finding focal or multifocal, poorly demarcated white matter demyelinating lesions on MRI.^107^, ^108^

Six cases of acute disseminated encephalomyelitis (5 adults and 1 child) have been described, with the disease starting 5 to 16 days after the initial fever‐arthralgia‐rash symptoms of chikungunya infection. Patients presented with a variety of neurological features, including headache; drowsiness; cranial nerve involvement such as facial nerve palsy, vertigo, nystagmus, and bulbar weakness; limb weakness; sensory disturbance; and urinary retention. MRI of the brain and/or spine suggested demyelinating pathology. All 6 were treated with intravenous methylprednisolone; the outcome varied between good clinical and radiological recovery^91^ and permanent neurological disability with confinement to a wheelchair and long‐term urinary catheterisation.^92^

### 7.4 Guillain‐Barré syndrome

Chikungunya‐associated peripheral neuropathy without CNS disease has been described for 72 patients in case reports or series, the majority of whom were described as having GBS. In one series of 4 patients with acute flaccid paralysis, no CSF or neurophysiology results were reported, making diagnosis difficult.^69^ Other causes of acute flaccid paralysis, such as anterior myelitis, have not yet been reported in association with chikungunya virus.

Two studies from Réunion Island showed an increased incidence of GBS following a large outbreak of chikungunya. One from the 2014 to 2015 outbreak reported 9 patients with chikungunya‐associated GBS, representing a 4‐ to 9‐fold increase in the island's annual GBS incidence.^70^ The increase in the incidence of GBS following the 2006 chikungunya virus outbreak on Réunion Island was estimated to be around 22% compared with the year before.^68^ A study from Martinique and Guadeloupe also showed an increase in incidence of GBS following the 2014 chikungunya virus outbreak, but to a lesser extent (2‐fold).^71^

Clinically, chikungunya‐associated GBS resembles GBS associated with other infections such as *Campylobacter jejuni*, presenting with symmetrical, bilateral flaccid weakness, often with paraesthesia and/or cranial nerve palsy.^109^ The 4 reports in the literature detailing the time interval between chikungunya infection and onset of neurological features describe a prodrome of 3 to 17 days, compatible with a parainfectious or postinfectious syndrome (Table 2).^67^, ^68^, ^73^

Unlike infections such as *C. jejuni*, which is associated with a more severe pure motor variant of GBS,^110^ chikungunya appears to be associated with the full range of GBS variants, as determined by neurophysiological studies, including disorders of motor and sensory axons, and myelin sheaths, sometimes in combination (see Table 2). Further investigation is required to determine risk factors and markers for the different GBS variants, including antiganglioside antibodies.

For the 36 cases where treatment was described, 28 (78%) received intravenous immunoglobulin, 2 (6%) intravenous immunoglobulin and plasma exchange, 4 (11%) intravenous methylprednisolone, and 2 (6%) plasmapheresis. Forty (87%) of the 46 cases for whom follow‐up data were available improved. In some cases, this was rapid; in most, it had occurred by 3 months.

### 7.5 Ocular complications

Although photophobia and conjunctivitis are associated with the acute phase of chikungunya infection,^111^ many later ocular complications have been described up to 12 weeks after infection, which may require emergency management. These include disease of the uvea, retina, and optic nerve. As well as inflammation, other pathologies have been described, including retinal detachment, intraretinal haemorrhage, and branch retinal artery occlusion (Table 2).^38^, ^75^ Our literature review found 78 cases of ocular complications of chikungunya infection.

In a retrospective study from India of 37 Indian patients with acute ocular manifestations and IgM‐confirmed chikungunya infection, uveitis was the most common diagnosis, occurring in 16 (43%) patients;^75^ 48 controls from the chikungunya‐endemic area, selected from patients attending the hospital for nonacute problems including cataract and refractive error, were all negative for chikungunya IgM. Recovery was variable—of the 26 patients followed up, the visual acuity improved in 11 (42%), remained the same in 12 (46%), and worsened in 3 (12%). Overall, of the 67 patients with ocular disease we identified for whom follow‐up data were reported, 46 (69%) recovered well, whereas 21 (31%) showed minimal or no improvement.

Although most of the serious ocular complications occur days to weeks after the acute chikungunya infection, in one report, 5 of 14 optic neuritis cases occurred simultaneously with the systemic disease onset.^74^ This suggests a more direct viral effect may be important, as well a postviral immune response.

Disease relapse has been reported in one report, which described bilateral uveitis and retinal detachment, with loss of visual acuity starting 4 days after symptoms of chikungunya infection.^82^ Having received a week's course of oral and topical steroids and recovered within 6 weeks, the patient re‐presented 3 months later with floaters and keratic precipitates; clinicians should be vigilant for relapse in such cases.

### 7.6 Disease affecting multiple components of the nervous system

As well as the distinct syndromes described above, chikungunya infection is associated with complex diseases involving multiple parts of the nervous system causing, for example, encephalomyelopathy (23 patients identified in our literature review), myeloneuropathy (24), and encephalomyeloneuropathy (24). Where follow‐up data were available, unlike in pure myelopathy, more of these patients had an unfavourable outcome (8 deaths, 4 no improvement) compared with those who improved (8).

### 7.7 Other

A handful of other neurological disorders have been associated with chikungunya, albeit in smaller numbers. A study from the Réunion outbreak described 32 patients presenting with behavioural changes including attention disorders, irritability, and memory issues.^37^ Other reports have described febrile seizures, isolated cranial nerve palsies, stroke, and hearing loss (Table 1), and one report (in press) describes the possibility of an association with chronic fatigue syndrome. Although it is difficult to definitively associate these isolated disorders with chikungunya infection, the full spectrum of chikungunya‐associated neurological diseases appears to be broad.

#### 7.7.1 Perinatally acquired neurological disease

Most of the evidence on chikungunya causing neonatal disease relates to transmission in the intrapartum period, rather than earlier in pregnancy. A wide range of severe manifestations has been described affecting neonates whose mothers had acute chikungunya infection near the time of delivery (Table 3). A case series from Colombia from 2014 to 2015 described 8 infants who required admission to an intensive care unit after contracting chikungunya infection perinatally.^31^ All mothers and neonates were PCR and IgM positive for chikungunya in serum; the neonates presented with severe diseases including meningoencephalitis, respiratory distress, sepsis, necrotising enterocolitis, myocarditis, and pericarditis.

**Table 3:** Reports of perinatally acquired neurological disease associated with chikungunya virus

Year of case(s), Location	No.	Evidence for Chikungunya	PP	CSF	Neurological Features	Treatment^a^ and Outcome
Perinatal encephalopathy (n = 35)						
2005‐2006, Réunion^37^	5	Ne: PCR CSF	…	…	Fulfilled International Encephalitis Consortium criteria for encephalitis	Cerebral palsy and blindness (1), poor neurodevelopmental performance (1)
2005‐206, Mayotte^36^	3	Ne&M: PCR/IgM CSF/ser	…	…	Unknown	Unknown
2005‐2006, Réunion^112^	4	Ne&M: PCR/IgM ser	3‐7 d	…	Seizures; EEG consistent with encephalitis	Survived
2010, India^113^	2	Ne&M: PCR ser	5 d	NAD	Altered sensorium, apnoeic seizures	Spastic diplegia, epilepsy, mental retardation
			3 d	NAD	Apnoeic seizures, lethargy	↓tone, cerebral palsy, ↓vision, mental retardation
2014‐2015, Colombia^31^	2	Ne&M: PCR/IgM ser	…	…	Unknown	Unknown
2014‐2015, El Salvador, Colombia, and Dom Rep^114^	12	Ne: PCR/IgM ser/CSF (10)	…	…	Unknown	Unknown
2015, Brazil^115^	1	Ne: PCR CSF, ser, urine, saliva	6 d	WCC N, prot↑	Seizures; abnormal MRI brain	*Anticonvulsants*. Improved at 17 d
2015, Honduras^52^	3	PCR ser	…	…	Unknown	Unknown
2016, India^116^	2	Ne&M: IgM ser	5 d	…	Dizygotic twins; both had seizures, required ventilation, thrombocytopenia; abnormal MRI brain	Both improved and discharged at 24 d
2016^c^, Brazil^117^	1	Ne: PCR CSF; M: IgM ser	4	WCC ↑, prot ↑	Prostration, lethargy, seizures, required ventilation, thrombocytopenia; abnormal MRI brain and EEG	Cerebral palsy, microcephaly, epilepsy at 1 y
Perinatal brain haemorrhage (n = 7)						
2005‐206, Réunion^105^	2	Ne&M: PCR/IgM CSF/ser	…	…	DIC, transient scattered parenchymal petechiae (1), cerebellar haematoma (1)	Unknown
2005‐2006, Réunion^97^	2	Ne&M: PCR/IgM CSF/ser	…	…	Unknown	Unknown
2005‐206, Réunion^112^	1	Ne&M: PCR and IgM ser	3‐7 d	…	Severe thrombocytopenia, cerebral haemorrhage	Survived
2015, Brazil^118^	1	Nil	4 d	NAD	Intraventricular bleed (cranial US), lethargy	Improved, discharged after 17 d
2012, India^119^	1	M: IgM ser; Ne: NAD	3	NAD	Lethargic, severe thrombocytopenia, focal bleeds basal ganglia, and subcortical areas	Fully recovered
Perinatal other (n = 18)						
2005‐2006, Réunion^97^	17^b^	Ne&M: PCR/IgM CSF/ser	…	…	Seizures (6); hypotonia (17)	Unknown
2005‐2006, Mayotte^36^	1	Ne&M: PCR/IgM CSF/ser	…	…	Hypotonia	Unknown

Abbreviations: …, data unavailable; CSF, cerebrospinal fluid; DIC, disseminated intravascular coagulation; Dom Rep, Dominican Republic; EEG, electroencephalogram; IgM, immunoglobulin M; M, mother; MRI, magnetic resonance imaging; N, normal; NAD, no abnormality detected; Ne, neonate; PCR, polymerase chain reaction; PP, onset of neurological disease days postpartum; prot, protein (↑, >0.4 g for adults, >1.5 g for neonates); ser, serum; WCC, white cell count (↑, >5 cells/μL); VEP, visual evoked potential.

a Treatments are in italics.

b At least 17 patients; unclear whether seizures and hypotonia were seen in the same or different patients.

c Patient had a subarachnoid haemorrhage and optic atrophy in addition to encephalitis.

One study from Réunion Island described 739 mothers who experienced symptoms of chikungunya infection during pregnancy, 39 of whom were symptomatic in the intrapartum period (between 2 d before and 2 d after delivery).^105^ Of these 39 mothers, all infants were asymptomatic at birth, but 19 developed acute disease a median 4 (range 3‐7) days after delivery, giving a vertical transmission rate of approximately 50% for mothers symptomatic in the intrapartum period. The initial clinical features in the affected neonates included fever, distress, poor feeding, petechiae, and a maculopapular rash. Nine cases (47%) were reported to have developed encephalopathy. Cerebrospinal fluid for all 9 showed normal biochemistry and white cell counts, and chikungunya PCR was detected in 5. Magnetic resonance imaging data from this study, combined with a later follow‐up study,^120^ show that amongst neonates developing encephalopathy or encephalitis after perinatally acquired chikungunya, severe white matter injury is well characterised in a 3‐stage pattern: cytotoxic brain oedema (ischaemia), vasogenic oedema (reperfusion), and mass reduction (demyelination).^105^, ^120^

In addition to these severe features, hypotonia has been described in 17 neonates with chikungunya infection from Réunion Island,^97^ as well as intracerebral haemorrhage secondary to clotting abnormalities.^105^, ^112^ Interestingly, neonatal spinal cord and peripheral nervous system diseases have not been reported.

With regard to prevention of transmission, no studies have found a protective effect of caesarean section.^105^, ^114^ The risk factors for vertical transmission are not understood, although in one study, the viral load of chikungunya in the placentas of the 19 transmitters was found to be significantly higher than in 13 nontransmitters.^105^ Interestingly, one of the transmitters gave birth to dizygous twins, of whom one acquired infection but the other did not.

In addition to overt diseases in some perinatally infected neonates soon after delivery, there is also evidence for impacts on longer‐term development. In one study comparing the neurocognitive function at approximately 2 years of age for 33 children with and 135 without perinatal chikungunya infection, significant differences in development quotients were found, including movement, coordination, language, and sociability.^120^ Importantly, even those infected at birth without obvious clinical features of neurological disease, such as encephalopathy, had significantly worsened neurocognitive function than uninfected children. Thus, the neurological effects of vertically transmitted chikungunya may not be obvious at birth, emphasising the importance of follow‐up of this cohort. The 12 cases with encephalopathy at birth showed still more severe developmental deficit, including cerebral palsy and microcephaly.

Maternal chikungunya virus infection earlier in pregnancy does not appear to affect the fetus; in the above study investigating 739 mothers with chikungunya infection, 700 were symptomatic outside of the intrapartum period, and none of these infants developed symptoms of chikungunya.^105^

Of note, in 3 of 7 miscarriages occurring before 22 weeks, chikungunya RNA was detected in amniotic fluid for all 3 and placenta and fetal brain for 2. However, no significant increase in antepartum fetal deaths was seen during the chikungunya outbreak as compared with previous years. Another study of 1400 mothers from Réunion Island^121^ found no effect of antepartum chikungunya infection on pregnancy outcomes. Together, these data would suggest that although antepartum congenital infection has been detected in miscarried fetuses, given the lack of epidemiological evidence for a causal association between infection and miscarriage, this may have been an incidental finding.

Zika virus is also now known to cause devastating neurological disease in neonates, which is of global concern.^122^ Whereas chikungunya appears to be most damaging around the time of birth, Zika has been associated with neurological sequelae in infections at all stages of pregnancy.^101^ Another important difference is in the initial presentation of neonatal disease—in perinatal chikungunya infections, fever‐rash and neurological symptoms are only seen at approximately 4 days postpartum. In the Zika congenital syndrome, the damage is done in utero; thus, neonates can be born with clear evidence of infection, such as microcephaly. Despite the magnitude of outbreaks throughout the tropics, there is a paucity of data on the effects of dengue virus in pregnancy. A recent large retrospective study from Brazil reported an increased risk of preterm birth associated with dengue infection, with no difference in the rate of congenital malformations.^123^ Adverse outcomes in neonates following perinatal dengue infection, such as thrombocytopenia, dengue haemorrhagic fever, and dengue shock syndrome, have been described in a handful of case reports and series in neonates^124^, ^125^, ^126^; but unlike chikungunya and Zika, apart from one case of anoxic encephalopathy,^127^ neurological complications have not been reported.

### 7.8 Pathophysiology

The mechanisms by which chikungunya virus affects the nervous system have not been fully elucidated. The following section discusses progress on some of the important unanswered questions, including how certain patients develop neurological disease after chikungunya infection and others do not, whether the virus acts directly or indirectly towards neurons and if the process differs in the CNS and peripheral nervous system, and the significance of the phylogenetic strain and factors driving placental transmission.

A study from India compared the cytokine profile for patients with and without neurological complications following chikungunya infection.^128^ Of those with neurological disease, 4 had encephalitis and 1 had “neuropathy.” Concentrations of 4 cytokines (TNF‐α, IFN‐α, IL‐6, and monokine induced by IFN‐γ) were found to be significantly higher in patients with neurological disease secondary to chikungunya, as opposed to uncomplicated chikungunya infection. However, the role of these cytokines in disease pathogenesis is still unclear.

Whether the virus affects the nervous system directly or indirectly via a triggered immune‐mediated effect is also unknown; scarce evidence argues for both. For the former, both RNA and virus have been isolated from CSF in severe CNS disease,^30^ consistent with direct neuroinvasion. Neurons, astrocytes, and oligodendrocytes (but not microglia) are susceptible to chikungunya infection in vitro; the former 2 cell types were shown to undergo apoptosis postinfection.^129^, ^130^ In vivo, subcutaneous inoculation in macaques resulted in morphological changes in astrocytes, including cell body hypertrophy and alteration in the pattern of branching of their primary processes.^131^ In relation to the latter, immune‐mediated hypothesis, this study also showed upregulation of *TLR2* in grey matter astrocytes, a gene associated with the innate immune response. The clinical neurological state of the macaques was not reported, which adds to the uncertainty of whether the immune response was protective or pathogenic in these cases. Another study that subcutaneously inoculated mice with chikungunya virus detected upregulation of *TLR3* in the brain, a gene that is also associated with the innate immune response.^132^ Amongst other clinical signs, these mice developed hind‐limb paralysis, dehydration, and weight loss, and 25% of them died after 1 week. Interestingly, pretreatment with polyinosinic:polycytidylic acid (a *TLR3* agonist and interferon inducer), which caused upregulation of proinflammatory cytokines, chemokines, antiviral genes, and IFN‐β, was protective clinically and promoted viral clearance from the brain, arguing for a protective innate and adaptive immune response, at least in CNS disease. In concordance, faster viral clearance after chikungunya infection was seen in wild‐type mice compared with a *TLR3*‐knockout model; this was thought to be secondary to increased antibody‐neutralising activity in the wild‐type mice.^133^ Chikungunya‐infected *TLR3*‐knockout mice had increased viral dissemination throughout the viscera, including the brain. Along with a U‐shaped pattern of age‐specific incidence, this critical role of *TLR3* is reminiscent of susceptibility to HSV encephalitis.

Patients diagnosed with myeloneuropathy or encephalomyeloneuropathy exhibit disease of both the CNS and peripheral nervous system. Given the association between chikungunya and GBS, it is not clear whether in these cases, there is one underlying pathological process involving both the CNS and peripheral nerves, or dual pathology, with a myelopathy ± encephalopathy centrally and GBS peripherally. For example, a case report from India described a 73‐year‐old man who, a week after chikungunya infection, was admitted with drowsiness, weakness, and absent reflexes and eventually died.^34^ His CSF and MRI showed evidence of CNS involvement, and an electromyogram showed a sensorimotor neuropathy. A brain autopsy showed subarachnoid haemorrhage, ischaemic changes, and small foci of demyelination without identification of viral inclusion bodies. Clearly, this patient had involvement of both CNS and peripheral nervous system at the same time, but it is not clear whether the same pathological process was responsible for both. Elucidation of these mechanisms may help to better guide management strategies.

Neurological disease secondary to chikungunya has been reported in areas with both ECSA (or ECSA‐diverged Indian Ocean lineage) and Asian strains, but whether these strains have differing neurovirulence is unknown. One study compared the effect of intracerebral inoculation of Asian and ECSA‐diverged strains in mice.^134^ Both spread within the brain to a similar extent, but the Asian strain was associated with higher mortality than the ECSA‐diverged strain. Upregulation of a gene associated with apoptosis was seen in the former, whilst antiapoptosis, antiviral, and CNS protective gene upregulation were seen in the latter. This potentially suggests a higher neurovirulence of the Asian strain, and comparative clinical data from countries such as Brazil, where both strains are circulating, will be useful.

On neonatal neurological disease, given that caesarean section is not protective, vertical transmission is unlikely to occur via the birth canal, as is the case in other neonatal infections such as herpes simplex.^135^ Furthermore, the placenta seems to act as a barrier to transmission, as one study reported (as unpublished data) that placental cells from infected neonates were negative when labelled with antichikungunya antibody.^105^ One hypothesis raised by the authors is that uterine contractions result in breaches of this placental barrier, allowing passive passage of the virus.

### 7.9 Management

There are currently no specific antiviral agents or vaccines for chikungunya virus.^136^ Various in vitro compounds active against chikungunya virus have been reported, including both direct‐acting and host‐targeting antivirals; however, most of these compounds have yet to find their way into in vivo models and clinical trials.^137^ Two of the few that have been tested clinically, chloroquine and ribavirin, are already widely used in the treatment of other diseases and have a known safety profile. However, chloroquine was found not to have any benefit for arthritic chikungunya when compared with a nonsteroidal anti‐inflammatory drug in a randomised clinical trial.^138^ Ribavirin, on the other hand, had promising results in a small case series of 10 patients with severe arthritis post–chikungunya infection.^139^ To the best of our knowledge, no antiviral has been evaluated in the management of chikungunya‐associated neurological disease, which therefore remains the same as that of neurological disease without associated chikungunya infection: for patients with encephalitis, those with a reduced Glasgow coma score (GCS) require assessment by intensive care specialists and may need intubation, ventilatory support, correction of electrolyte abnormalities, management of raised intracranial pressure, and enhancement of cerebral perfusion pressure.^140^ In patients with myelitis, corticosteroids are the standard first‐line treatment, despite the lack of trial evidence for their use in this scenario.^141^ The management of GBS focuses on immunotherapy with intravenous immunoglobulin or plasma exchange, and ventilatory support if the innervation of respiratory muscles is affected.^109^

Although not yet commercially available, it is hoped that a vaccine for chikungunya is on the horizon. Two phase 1 clinical trials have shown a good safety and immunogenicity profile to date.^142^, ^143^ A recent study that tested an insect‐specific alphavirus as the vaccine platform found promising results in mice and macaques, including immunogenicity after a single dose.^144^

## 8 CONCLUSION

Neurological disease associated with chikungunya virus is being reported increasingly, in part because of the recent introduction of the virus to the South American population and associated large outbreaks. Clinicians and public health officials globally face challenges from the wide range of associated neurological disease and the complicating factor that dengue and Zika viruses are transmitted by the same mosquito vectors and have broadly similar epidemiology. In endemic areas, chikungunya virus should be tested for in all patients presenting with acute neurological disease and all mothers presenting with fever, arthralgia, or rash; neonates with suspected infection should be followed up for at least 2 years for evidence of neurodevelopmental delay, regardless of the initial presentation (Table 4). Future challenges include understanding the full scope of chikungunya neurological disease, in both neonatal and adult infection, and their underlying pathophysiological mechanisms. It is hoped that new direct therapeutic and vaccine candidates, some of which have shown promise in early studies, will augment the current supportive management strategies.

**Table 4:** For the clinician—summary

Adults and Children (Transmission Directly Via Mosquito Bite)	Neonates (Vertical Transmission)
Patients in areas endemic for chikungunya, Zika, or dengue presenting with an acute neurological disorder should be investigated for all 3 arboviruses	Neonates born to mothers experiencing symptoms of chikungunya infection near the time of delivery require admission and observation for signs of vertical transmission for at least 7 d postpartum, as they may be asymptomatic for the first few days of life
Encephalitis is the most commonly reported neurological complication associated with chikungunya; encephalitis has a worse prognosis than encephalopathy alone; a CSF pleocytosis is not always seen	Neonates born to mothers infected outside of the peripartum period are usually unaffected by chikungunya virus
In myelitis associated with chikungunya, CSF pleocytosis and magnetic resonance imaging changes are not always seen	Caesarean section does not appear to be protective in vertical transmission of chikungunya
Guillain‐Barré syndrome associated with chikungunya follows a similar course compared with other infections such as *Campylobacter jejuni*; most patients recover after immunomodulatory treatment	Neonates infected with chikungunya should be followed up for at least 2 y, regardless of symptoms in the first week of life; the neurodevelopment of those without clinical encephalopathy at birth can still be affected
Disease of both the central and peripheral nervous systems in the same patient can be seen in association with chikungunya infection	
Ophthalmological complications associated with chikungunya have been reported both at the time of infection and up to 12 wk after; some reports describe treating with steroids, recovery is variable	
Following chikungunya infection, complications of other organs can also occur at the same time as disease of the nervous system; such cases should be managed using a multidisciplinary approach	
There is currently no available antiviral treatment or vaccine for chikungunya	

### AUTHOR CONTRIBUTION

All authors performed the literature search and reviewed the included articles. R.M. wrote the initial draft, and all authors commented on and edited the manuscript.

## CONFLICT OF INTEREST

We declare no competing interests.
